# Cutaneous and Systemic Psoriasis: Classifications and Classification for the Distinction

**DOI:** 10.3389/fmed.2021.649408

**Published:** 2021-10-13

**Authors:** Bing-Xi Yan, Xue-Yan Chen, Li-Ran Ye, Jia-Qi Chen, Min Zheng, Xiao-Yong Man

**Affiliations:** Department of Dermatology, Second Affiliated Hospital, Zhejiang University School of Medicine, Hangzhou, China

**Keywords:** psoriasis, cutaneous and systemic, classification criteria, therapy, diagnosis, biologics

## Abstract

Psoriasis is a chronic multisystem inflammatory disease that affects ~0.1–1.5% of the world population. The classic cutaneous manifestation of psoriasis is scaly erythematous plaques, limited or widely distributed. Moreover, psoriasis could be associated with comorbidities like psoriatic arthritis, metabolic syndrome, diabetes, cardiovascular disease, nephropathy, bowel disease, and brain diseases. In this review, we suggest that psoriasis should be classified as cutaneous psoriasis or systemic psoriasis and propose the classification for distinction. This would help to better understand and manage psoriasis.

## Introduction

Psoriasis is a chronic systemic inflammatory disease that affects about 0.1–1.5% of the population worldwide (1). Proinflammatory cytokines such as interleukin-23 (IL-23), IL-17, and tumor necrosis factor α (TNF-α) play critical roles in the initiation and maintenance of psoriasis (2, 3). Approximately 36% of patients with psoriasis have a family history of psoriasis, and multiple genetic susceptibility loci have been identified (4, 5). Psoriasis could be triggered by a variety of extrinsic and intrinsic risk factors (6).

The incidence of psoriasis varies greatly around the world and is related to races, geographic locations, and environment (7).

The classic cutaneous presentation of psoriasis is scaly erythematous plaques, localized or widely distributed (8). Up to 30% of patients with psoriasis could develop psoriatic arthritis (9). Moderate-to-severe psoriasis is associated with an increased risk of metabolic syndrome and cardiovascular disease (10–12).

Diagnosis of psoriasis is mainly made upon clinical findings and a skin biopsy is rarely required (13). However, immunogenic evidence challenges the traditional taxonomy of psoriasis and suggests the reclassification of psoriasis into several subtypes. Sustained remission is an ultimate goal in the management of psoriasis, especially for moderate-to-severe psoriasis. Personalized systemic medicine such as biologics, could not only treat psoriatic lesions but also prevent or improve systemic comorbidities (14). Herein, we propose new classifications of psoriasis and classification for distinction.

## Classifications of Psoriasis

Psoriasis vulgaris is the most common phenotype, affecting ~85–90% of patients with psoriasis (3, 15). The most commonly affected areas of the lesions include the extensor surfaces of elbows and knees, the sacral region, and the scalp, though lesions may involve any part of the skin (1). Furthermore, growing evidence suggests that compared to the general population, patients with psoriasis have a higher prevalence in other chronic and serious health diseases, including arthritis, metabolism disease, diabetes, cardiovascular diseases, hypertension, depression or anxiety, liver disease, Crohn's disease, and lymphoma or other cancers (2, 13, 15). Therefore, we suggest psoriasis may be classified into cutaneous psoriasis and systemic psoriasis.

### Cutaneous Psoriasis

#### Plaque Psoriasis (Psoriasis Vulgaris)

The typical lesions of plaque psoriasis are erythematous, scaly, and well-demarcated plaques (2). The psoriasis area severity index (PASI) is an index used to determine the severity of psoriasis. It combines the severity (erythema, induration, and desquamation) and the percentage of affected areas (16). Lesions begin as erythematous papules that gradually enlarge into rich red (also referred to as “salmon pink”) plaques. The shape of plaque and the amount of scaling are variable, but most lesions are covered by silvery white scaling. When gently scraping the surface, scales fall off like candle wax (wax-spot phenomenon); this is a sign of the parakeratosis and hyperkeratosis of the epidermis. When plaques are scraped deeper, a wet smooth layer can be revealed referred to as the “last membrane phenomenon.” On the background of the erythematous membrane, pinpoints-like bleeding foci appear known as “Auspitz's sign” (3).

The clinical features of scalp psoriasis vary from intermittent mild erythematous scaly plaques to total scalp involvement, usually beyond the hairline giving the appearance of bundled hair (13).

Nail psoriasis mostly demonstrates as nail pitting. Other presentations range from oil drop discoloration, splinter hemorrhaging of the nail bed to crumbling or loosening of the nail plate (17). Of note, it is an important predictor for psoriatic arthritis (PsA) (18). An estimated 50% of patients have nail psoriasis at the time of diagnosis of psoriasis, which contributes to a greater social burden and worsens the quality of life in these patients (13, 19).

#### Guttate Psoriasis

Guttate psoriasis is more common in children and adolescents than adults and is usually triggered by streptococcal infection. Patients were classically present with numerous, scaly, small “drop-like” papules and plaques that are 0.3–0.5 cm in diameter (2). Itch is of various levels and intensity. One-third of guttate psoriasis would develop into chronic plaque psoriasis in later life (20–22).

#### Pustular Psoriasis

Pustular psoriasis is characterized by white sterile pustules, either in generalized or localized distribution. The typical presentation is an eruption of superficial pustules with an erythematous base (23). Pustular psoriasis is further divided into generalized pustular psoriasis (GPP) and localized pustular psoriasis. Localized pustular psoriasis includes palmoplantar pustulosis (PPP) and acrodermatitis continua of Hallopeau (ACH) (23).

##### Generalized Pustular Psoriasis

Generalized pustular psoriasis is a neutrophilic autoinflammatory skin disease characterized by widespread sterile pustules, which can occur with or without a history of plaque psoriasis (24). GPP is often acute onset on formal psoriatic lesions or normal skin, which may be accompanied by systemic inflammation (25). Superficial aseptic small pustules appear rapidly. Densely distributed pustules often expand and coalesce, forming lakes of pus (2). Acute GPP is often associated with systemic symptoms such as chills, high fever, malaise, anorexia, nausea, and severe pain (26). Other presentations could be geographic tongue, thick and turbid nail plates, and subungual pustules (26). Generally, the pustules dry out and form crusts, and high fever relives at the same time. But pustules and high fever may recur periodically (20). Acute GPP flares may be triggered by medication withdrawal (especially systemic withdrawal of corticosteroids), infections, stress, medication, and pregnancy, causing a dramatic reduction in quality of life (27). Acute GPP could lead to mortality without appropriate treatment because of accompanied infections and multiple systemic function failures.

##### Palmoplantar Pustulosis

Palmoplantar pustulosis is a rare, chronic, recurrent inflammatory disease that affects palms and/or soles with sterile, symmetrically distributed, erupting pustules that appear on an erythemato-squamous background (28). Pustules are more likely to occur in the middle and inner part of palms and/or soles and may extend to the dorsal aspect of hands and fingers (or feet and toes) (2). It could persist for years and usually be resistant to treatment, with periods of partial or total remission interrupted by recurrent exacerbations (28). Nails are often affected, presented with pitting, lateral grooves, longitudinal crests, nail turbidity, nail stripping, and empyema (20).

##### Acrodermatitis Continua of Hallopeau

Acrodermatitis continua of Hallopeau is a rare, sterile, macroscopically visible pustule affecting the nail apparatus of one or more digits (29). ACH manifests with tender pustules and underlying erythema on the tip of a finger, sometimes on the toe (30). Nails are always involved in ACH; if there is no nail involvement, then alternative diagnoses such as PPP should be considered (29). Pustules may also be present on the nail bed (under the nail plate). When patients were under unsuccessful treatment, severe complications could appear such as ongchoptosis and osteolysis, which further reduce the quality of life in these patients (30).

#### Erythrodermic Psoriasis

Erythrodermic psoriasis (EP) is a rare and severe variant of psoriasis, which presented with generalized (usually involved 90% or more of body surface area) erythema, edema, pruritus, scaling, exudative lesions, and palmoplantar or diffuse desquamation (31). EP is always accompanied by systemic symptoms such as chills, fever, dehydration, lymphadenopathy, gastrointestinal malaise, rarely high output heart failure, and cachexia (31). Due to the dysfunction of the skin barrier, EP patients can present with high possibilities of severe systemic infection and sepsis, which occasionally could be life-threatening (32). The course of EP is long and easy to relapse (2, 20).

#### Inverse Psoriasis

Inverse psoriasis (IP) is also named intertriginous or flexural psoriasis (33). The lesions typically present as smooth, moist, scaly-less, dark-red patches in the folds or rubbing areas, including the inguinal folds, axillaes, inframammary folds, anogenital areas, umbilicus, and retro-auricular areas such as hip groove, armpit, groin, and under the breast; retro-auricular regions and glands. It is common to see patients with IP have typically plaque psoriasis lesions localized in other body areas (33).

#### Stages of Cutaneous Psoriasis

The cutaneous psoriasis process could be categorized into three stages: progressive stage, stationary stage, and regressive stage.

##### Progressive Stage

New inflammatory lesions continue to appear, presenting as erythematous thick-scaly plaques. A local cutaneous trauma caused by acupuncture, scratching or surgery may lead to typical psoriatic lesions. This phenomenon is named isomorphism or the KÖbner phenomenon (34).

##### Stationary Stage

Lesions become stable, demonstrating as light-red scaly plaque, and new lesions barely appear.

##### Regressive Stage

Lesions are flattening of infiltration with slight or no scaling. Some lesions could leave hypopigmentation or pigmentation marks.

#### The Severity of Cutaneous Psoriasis

Cutaneous psoriasis severity categories are important for clinicians to not only make treatment decisions but also to identify eligibility criteria for clinical studies (35). Combining body surface area (BSA), psoriasis area and severity index (PASI), and investigator's global assessment (IGA), cutaneous psoriasis are divided into three categories: mild, moderate, and severe (36) (as shown in Table 1).

**Table 1:** The severity of cutaneous psoriasis.

**Mild**	**Moderate**	**Severe**
BSA <3%	3% ≤ BSA <10%	BSA≥10%
PASI <3	3 ≤ PASI <10	PASI≥10
IGA=1	IGA=2	IGA=3/4

*BSA, body surface area; PASI, psoriasis area and severity index; IGA, investigator's global assessment*.

### Systemic Psoriasis

In addition to psoriatic lesions, other systemic diseases can occur first, simultaneously or sequentially. Evidence indicates that psoriasis is an important systemic inflammatory disease (2, 13, 37, 38), sharing manifestations with other chronic inflammatory diseases (15). After treatment, psoriatic lesions improve and the associated systemic symptoms generally improve (39). Herein, we propose the name “systemic psoriasis” to emphasize the characteristic of systemic-effect of psoriasis. According to different combordities, a personalized clinician team should be referred to make a correct diagnosis and provide optimized treatment.

#### Psoriatic Arthritis

Nearly 30% of patients with psoriasis progress to psoriatic arthritis (PsA) (40). In addition to psoriatic lesions, PsA may affect any joint in the body, from large joints like elbows and knees, to small joints like fingers and toes, the spine and the sacroiliac joints (41). It is progressive and could cause the affected joints to become swollen and painful resulting in either oligoarticular or polyarticular arthritis, restricting mobility, and resulting in joint destruction and deformity in severe cases (2, 42). It is important to note that the blood test of the rheumatoid factor is often negative (2, 42). X-ray characteristic features of PsA include soft tissue swelling, varying degrees of joint erosions, joint space narrowing, and osseous proliferation, including periarticular and shaft periostitis, as well as osteolysis (43). Up to 90% of patients with PsA have nail psoriasis involvement (44, 45).

Psoriatic arthritis may be further divided into several subtypes: distal subtype (damage of proximal and distal interphalangeal joints of the hands and feet), oligoarthritis (arthritis involving four joints at most), polyarthritis (arthritis affecting five or more joints), arthritis mutilans (resorption and shortening of finger bones), axial/ankylosing spondylitis, enthesitis, and dactylitis (46).

#### Psoriasis With Metabolic Syndrome

Moderate to severe psoriasis is frequently associated with metabolic disorders, especially metabolic syndrome (MetS) (47). MetS could combine various interrelated metabolic disorders including obesity, insulin resistance, dysglycemia, atherogenic dyslipidemia, and hypertension (10–12, 14).

#### Psoriasis With Cardiovascular Disease

Psoriasis is an independent risk factor in cardiovascular diseases, including hypertension, hyperlipidemia, major adverse cardiovascular events, and myocardial infarction (42, 48–51).

#### Psoriasis With Nephropathy

In addition to psoriasis cutaneous lesions, psoriasis patients could have kidney damage, or confirmed immune-related kidney disease (42, 52–54). Psoriasis is considered to be an independent risk factor of chronic kidney disease and end-stage renal disease (55).

#### Psoriasis With Bowel Disease

Patients with inflammatory bowel disease (IBD), including Crohn's disease and ulcerative colon disease, share similarities in genetic susceptibilities and immune-mediated inflammation with psoriasis (56–59). There are significant bi-directional associations between psoriasis and IBD (58).

#### Psoriasis With Brain Diseases

Psoriasis has an extensive emotional and psychosocial effect on patients. Patients with psoriasis are accompanied by depression/mania, multiple sclerosis, or other mental symptoms, and may also have a significantly decreased quality of life and psychological burden including anxiety, depression, and suicidal thoughts and behavior (60–62).

#### Psoriasis With Pulmonary Disease

Interstitial lung disease or chronic obstructive pulmonary disease (COPD) can be seen in some patients with psoriasis (63–65).

#### Psoriasis With Liver Disease

Psoriasis is commonly accompanied by non-alcoholic fatty liver disease (66), liver fibrosis (67), or abnormal liver function.

#### Psoriasis With Uveitis

Uveitis is a known ophthalmologic manifestation of inflammation of the iris, ciliary body, and choroidal tissues. It is characterized by redness of the conjunctiva, eye pain, blurred vision, and flying mosquitoes. A significantly increased risk of both prevalent and incident uveitis is observed among patients with psoriasis (68).

#### Psoriasis With Lupus Erythematosus

It is rare for patients with psoriasis to have lupus erythematosus at the same time (14). Serologically positive lupus erythematosus patients are associated with psoriasis or are induced with psoriasis-treatment drugs.

#### Psoriasis With Malignancy

Psoriasis has also been associated with a low but elevated risk of malignant tumors of the skin or internal organs (42).

## Classification Criteria for Psoriasis

Based on the clinical manifestations of psoriasis, we propose a new classification criteria for psoriasis (as shown in Table 2): cutaneous psoriasis and systemic psoriasis, according to the following classification criteria (Table 2). A psoriatic family history should be taken into account. If there is still doubt about the diagnosis, a simple punch biopsy can be performed.

**Table 2:** Classification criteria for psoriasis from CPRWERG.

**Medical indexes**	**Symptom**	**Weight/score**
**Cutaneous psoriasis: Score 4 or above**		
Silver white scales	White scales, falling off in layers after scratching, called candle wax phenomenon	1
Film phenomenon	Scraping off the silvery white scales, a shiny reddish translucent film on the surface of the lesions exposes	2
Auspitz's sign	Scraping continues deeper, the lesions will show punctate spots of bleeding	3
Hyperkeratosis and parakeratosis	Thickened cornified layer and cell nuclei present in the cornified layer	1
Highly reduced or absent Granular Layer	Thinning or absence of a granular layer	1
Acanthosis with elongated rete ridges	Thickening of viable epidermal layers	1
Angiogenesis	Dilated and contorted blood vessels reach into the tips of the dermal papillae	1
**Pustulosa psoriasis: Score 5 or above**		
Cutaneous psoriasis (Prerequisites)	Current or previous history of cutaneous psoriasis or family history of psoriasis	3
Fever	Temperature≥ 37.5°C	1
Pustule	On the basis of generalized erythema, multiple aseptic pustules, densely distributed or fused into flakes	2
	Pustules are easy to rupture and form flakes	1
Subcorneal pustulosis	The pathological manifestation is the aggregation of neutrophils to form subcorneal pustulosis, Kogoj's spongy abscess	1
**Erythroderma psoriasis: Score 5 or above**		
Cutaneous psoriasis (Prerequisites)	Current or previous history of cutaneous psoriasis or family history of psoriasis	3
Skin lesion area is over 90%	Diffuse flushing, infiltration and swelling of the skin all over the body, accompanied by a large number of bran-like scales, with flaky normal skin island	2
Fever	Temperature ≥ 37.5°C	1
**Systemic psoriasis: Score 6 or above**		
Cutaneous psoriasis (Prerequisites) (3 scores)	Current or previous history of cutaneous psoriasis or family history of psoriasis	3
Psoriatic arthritis (46, 69) (≥3 scores)	Finger (toe) nail changes	1
	Rheumatoid factor negative	1
	Finger (toe) arthritis	1
	Joint pain lasting more than 1 month	1
	Imaging showed new bone formation around the joint	1
Psoriasis with metabolic syndrome (10) (≥3 scores)	Increased waist circumference (Male>40 inches (102 cm) Female>35 inches (88 cm)	1
	Hyperglycemia: fasting blood glucose: ≥ 6 mmol/l (100 mg/dL), and/or have been diagnosed with diabetes and treated	1
	Hypertension: systolic/diastolic blood pressure ≥130/85 mmHg, and/or diagnosed with hypertension and treated	1
	Fasting triglyceride ≥ 150 mg/dL	1
	HDL cholesterol male <40, female <50 mg/dL	1
Psoriasis with diabetes (70) (≥3 scores)	HbA1c ≥ 6.5 % (≥ 48 mmol/mol)	3
	Random blood glucose≥ 200 mg/dl (≥ 11.1 mmol/l)	3
	Fasting blood glucose ≥ 126 mg/dl (≥ 7.0 mmol/dl)	3
	Two hours postprandial blood glucose (2hPBG) ≥ 200 mg/dl (≥ 11.1 mmol/l)	3
Psoriasis with cardiovascular disease (42) (≥3 scores)	Coronary heart disease	3
	Atherosclerosis	3
	Hyperlipidemia	3
	hypertension	3
	Ischemic heart disease	3
Psoriasis with nephropathy (≥3 scores)	Urine protein 1 + and above or 0.5 g/24 h	3
	Hematuria or erythrocyte cast	1
	Abnormally increased Urea nitrogen	1
	Abnormally elevated creatinine	3
Psoriasis with bowel disease (≥3 scores)	Confirmed Crohn's disease	3
	Confirmed ulcerative colitis	3
Psoriasis with brain disease (≥3 scores)	Depression/mania	3
	Psychiatric symptoms	3
	Multiple sclerosis	3
Psoriasis with pulmonary disease (42) (≥3 scores)	Interstitial lung disease	3
	COPD	3
Psoriasis with liver disease (≥3 scores)	Liver fibrosis	3
	Abnormal liver function (needs to exclude drugs)	3
	cholecystitis	3
Psoriasis with uveitis (≥3 scores)	Inflammation of iris, ciliary body or choroid tissue	3
Psoriasis with lupus erythematosus (≥3 scores)	ANA positive	1
	Anti-ds-DNA or anti-Smith antibody Positive	2
	EULAR/ACR Classification criteria for systemic lupus erythematosus (71)	10
Psoriasis with malignant tumors (42) (≥3 scores)	Squamous cell carcinoma/basal cell carcinoma	3
	Malignant melanoma	3
	Cutaneous T cell lymphoma	3
	Hematological malignancies	3
	Other malignant tumors	3

*CPRWERG, The Chinese Psoriasis Real-World Evidence Research Group*.

## Therapy for Psoriasis

Choice of treatment depends on many factors, mainly including patients and agendas, the former including the onset age, the duration, extent of disease, site of the lesions, the age of the patient, the type of psoriasis (cutaneous or systemic), pregnant or not, infection (especially tuberculosis or hepatitis B) or not, medical insurance covered or not, past therapy history and treatment willingness of the patients. The latter includes the efficacy, safety, price, response time, maintenance, frequency, and resistance of the drug. For moderate-to-severe cutaneous psoriasis and systemic psoriasis, biological therapies could be considered (37). Biologics could target specific molecules which could be essential in psoriasis pathogenesis, such as tumor necrosis factor (TNF) α, interleukin (IL)-17, IL-23, and IL-12 (72). Cutaneous psoriasis may resolve entirely after appropriate therapies targeting specific immune molecules.

**Figure 1:**
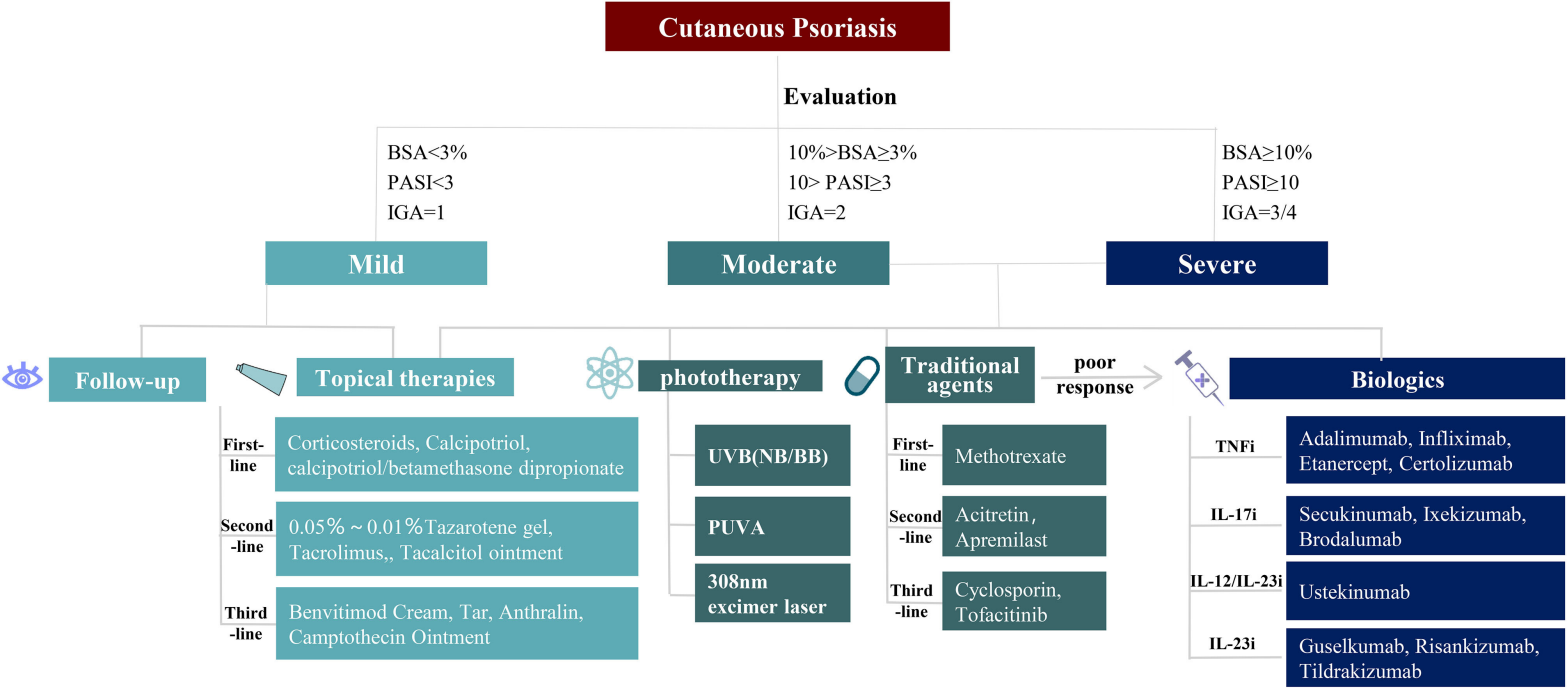
Therapies for cutaneous psoriasis. Different treatments for mild, moderate, and severe patients with cutaneous psoriasis. For mild cutaneous psoriasis, follow-up and topical therapies are advised choice. For moderate to severe cutaneous psoriasis, topical therapies, phototherapy, traditional agents, and biologics can be selected one by one. *BSA*, body surface area; *PASI*, psoriasis area and severity index; *IGA*, investigator's global assessment; *UVB*, ultraviolet radiation b; *NB*, narrow bound; *BB*, broad bound; *PUVA*, psoralen plus ultraviolet radiation a.

We suggest therapeutics for mild, moderate, and severe adult cutaneous psoriasis as shown in Figure 1. For mild cutaneous psoriasis, topical agents are recommended, or just a wait-and-see approach. For moderate and severe cutaneous psoriasis, topical agents, phototherapy, systemic non-biological therapy, and/or biologics could be chosen (14, 37, 73).

**Figure 2:**
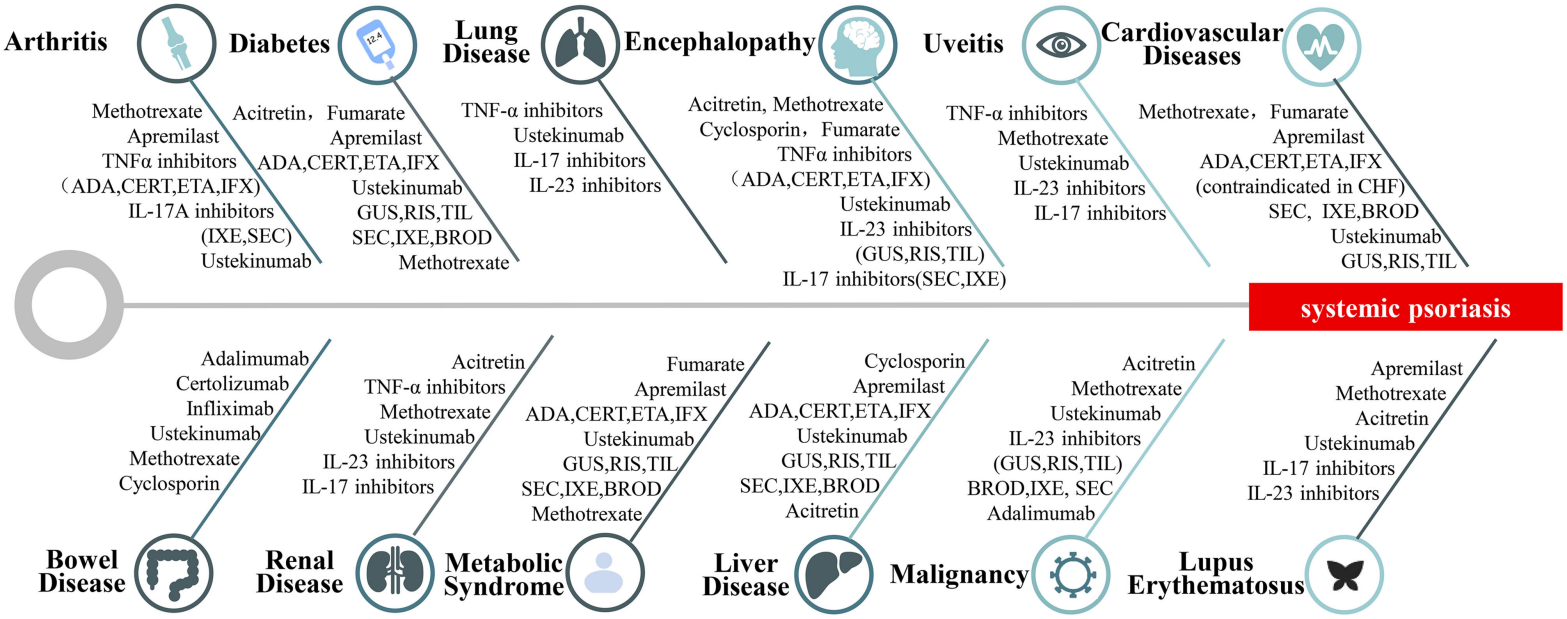
Therapies for systemic psoriasis. Selection of treatments for different types of systemic psoriasis includes psoriatic arthritis, psoriasis with diabetes, psoriasis with pulmonary disease, psoriasis with brain diseases, psoriasis with uveitis, psoriasis with cardiovascular disease, psoriasis with bowel disease, psoriasis with nephropathy, psoriasis with metabolic syndrome, psoriasis with liver disease, psoriasis with malignancy, psoriasis with lupus erythematosus.

Comorbidities could influence the treatment strategy (14). Figure 2 lists selected therapeutics for adult systemic psoriasis with comorbidities (74–77). Treatment should be tailored to meet the needs of patients (Figure 2). It is recommended to select treatments from top to bottom for each category of systemic psoriasis.

**Figure 3:**
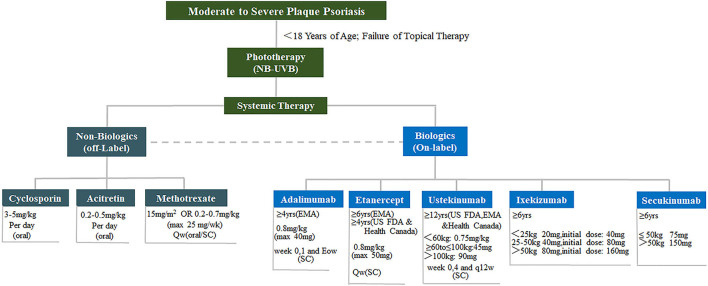
Therapies for pediatric psoriasis. Treatment of moderate to severe pediatric psoriasis. Non-biologics include cyclosporin, acitretin and methotrexate. Biologics include adalimumab, etanercept, ustekinumab, and ixekizumab. *NB-UVB*, narrow bound ultra violet B light.

For the therapy of pediatric psoriasis for patients under 18 years, AAD/NPF jointly published a comprehensive review recently (38). Briefly, pediatrics failure to topical therapy may undergo phototherapy and systemic therapy (Figure 3) (78). Long-term maintenance at the lowest effective dose with the least toxic therapy is the preferred approach (38). Till now, four biologics, etanercept, adalimumab, ustekinumab, and ixekizumab have been approved by FDA or EMA for pediatric psoriasis, which may be considered as first-line systemic agents (38).

## Conclusions

Psoriasis can be classified as cutaneous psoriasis and systemic psoriasis. Cutaneous psoriasis can be subdivided into plaque, inverse, erythrodermic, pustular, and guttate forms. In addition to cutaneous manifestations, systemic cormobidities may present in systemic psoriasis. Psoriasis patients should undergo a complete history query and a thorough physical examination, including joints, and other systemic diseases. Optimal management of psoriasis depends on the type of psoriasis and the severity of the disease.

## Author Contributions

B-XY, X-YC, and L-RY: conceptualization, formal analysis, resources, and writing the original draft. J-QC, MZ, and X-YM: conceptualization, funding acquisition, and writing review and editing. All authors contributed to the article and approved the submitted version.

## Funding

This study was supported by the National Natural Science Foundation of China (No. 81930089).

## Conflict of Interest

The authors declare that the research was conducted in the absence of any commercial or financial relationships that could be construed as a potential conflict of interest.

## Publisher's Note

All claims expressed in this article are solely those of the authors and do not necessarily represent those of their affiliated organizations, or those of the publisher, the editors and the reviewers. Any product that may be evaluated in this article, or claim that may be made by its manufacturer, is not guaranteed or endorsed by the publisher.
